# The Effects of Booking Status on the Outcome of Infants of ≥32 Weeks Gestational Age Admitted to the Neonatal Intensive Care Unit in a Tertiary Academic Center

**DOI:** 10.7759/cureus.31020

**Published:** 2022-11-02

**Authors:** Saad S Almarri, Yahya A Alzahrani, Mousab S Alsudais, Maha Bamehrez, Raseil K Alotaibi, Bushra S Almalki, Asail S Almukhles, Heidi Al-Wassia

**Affiliations:** 1 Pediatrics and Neonatology, Saudi Ministry of Health, Taif, SAU; 2 Pediatrics, King Abdulaziz University Hospital, Jeddah, SAU; 3 Pediatrics and Neonatology, College of Medicine, Majmaah University, Majmaah, SAU; 4 Neonatology, King Abdulaziz University, Jeddah, SAU; 5 Medical School, King Abdulaziz University Hospital, Jeddah, SAU; 6 Pediatrics and Neonatology, King Abdulaziz University, Jeddah, SAU

**Keywords:** prenatal care, pregnant women, infant outcome, maternal booking, antenatal care

## Abstract

Introduction

Antenatal care (ANC) is a systematic examination and follow-up of pregnant women that involves education, counseling, screening, and treatment of any complications encountered. ANC is an essential measure that significantly decreases devastating maternal and fetal outcomes. This study aimed to explore the maternal and fetal outcomes of mothers who did and did not book follow-ups and had their newborns admitted to the neonatal intensive care unit (NICU) at the King Abdulaziz University Hospital (KAUH) in Jeddah, Saudi Arabia.

Methodology

We conducted a cross-sectional study between January 1, 2021, and January 1, 2022, at KAUH in Jeddah, Saudi Arabia. Data were collected from electronic medical records and paper documents. Maternal demographic and pregnancy information were collected in addition to neonatal outcomes.

Results

The study included 186 participants, with a median maternal age of 32 years (interquartile range (IQR) 27-36). Cesarean section was the predominant mode of delivery (67.2%), with a median gestational age at birth of 36 weeks (IQR 34-38). Most women (69.4%) booked follow-ups, while 40.3% developed chronic comorbid conditions during pregnancy. The newborn sex ratio was nearly even between males and females, with a median birthweight of 2325 g (IQR 1740-2900) and median Apgar scores of 7 (IQR 5-9) and 9 (IQR 8-10) at 1 and 5 min, respectively. Jaundice was the most common postnatal complication (51.6%), followed by hypoglycemia (18.8%), while 23.7% of babies had congenital anomalies. There was a significant association between booking status and nationality, maternal age, cesarean section, maternal comorbid conditions, the outcome of multiple gestations, and postnatal complications, including jaundice and hypoglycemia. Decreasing maternal age (odds ratio (OR) 0.755, 95% confidence interval (CI) 0.585-0.974) and Apgar score at 5 min (OR 0.096, 95% CI 0.012-0.795) were the only significant predictors of fetal mortality.

Conclusion

The study revealed suboptimal adherence to ANC among pregnant women with newborns admitted to the NICU, along with poorer maternal and fetal outcomes, with respect to neonatal jaundice, hypoglycemia, and the need for resuscitation.

## Introduction

Antenatal care (ANC) refers to the meticulous systematic examination and follow-up of pregnant women involving education, counseling, screening, and treatment to ensure the best possible health of pregnant women and fetuses [1]. Pregnant women should visit their healthcare provider at least four times during pregnancy for routine checkups and additional appointments if needed, with prenatal care being provided by either a doctor, midwife, or nurse practitioner [2,3].

The importance of prenatal care in pregnant women has long been established and has a crucial link to maternal and fetal outcomes. Pregnant women can be diagnosed and treated for various health problems through prenatal care, preventing the development of complications during pregnancy and helping them plan their delivery, thus allowing them to have healthy babies [4,5].

It is vital to note that prenatal care focuses not only on the physical health of pregnant women but also on their emotional and psychological well-being, ensuring a holistic approach to care during pregnancy. This can help prevent depression and anxiety among pregnant women, which may be detrimental to the health of their newborns [6].

Previous evidence has indicated that poor fetal outcomes in terms of perinatal morbidity and mortality are associated with poor ANC adherence, which can be linked to factors such as preterm delivery and low birthweight. In addition, studies have shown that sufficient ANC attendance decreases the likelihood of low birthweight and admission to the neonatal intensive care unit (NICU) [7-11].

Suboptimal adherence to ANC visits is a critical problem. Although it is thought that most women attend at least one appointment, non-booking (pregnant women not attending ANC visits vs. booked mothers who attend regular and a sufficient number of ANC visits) has deleterious repercussions on the health of the mother and the fetus [12]. This study aims to explore the maternal and fetal outcomes of booked and unbooked mothers who had their newborns admitted to the NICU at the King Abdulaziz University Hospital (KAUH) in Jeddah, Saudi Arabia.

## Materials and methods

Study design

The study was approved by the institutional review board of the KAUH (reference number: 336-22). This study is a retrospective chart review of all infants born at ≥32 weeks' gestation and admitted to the NICU. This retrospective study was cross-sectional and analytical, and was conducted between January 1, 2021, and January 1, 2022, at the KAUH in Jeddah, Saudi Arabia. The NICU has level-II and -III neonatal intensive care beds and an average number of 2,800 deliveries per year in the last four years, with an admission rate of approximately 12.93%.

Study sample size and population

In this study, the targeted population was all infants born at ≥32 weeks’ gestation and admitted to the NICU at the KAUH during the study period. Infants not admitted to the ICU were excluded.

Data Collection and Definition of Variables

Maternal and infant characteristics were retrieved from electronic medical records and paper charts. Patient records from January 1, 2021 to January 1, 2022 were reviewed.

Maternal characteristics

The following data on the mothers were collected: nationality, age, gravidity, method of delivery, gestational age (based on the last menstrual period or pregnancy testing, prenatal ultrasound, or both), booking status, chronic diseases such as diabetes mellitus (DM) and hypertensive disorders, and antenatal steroid therapy.

Infant characteristics

The following data on the infants were collected: sex, birthweight, multiple pregnancies (twins or triplets), APGAR scores calculated at 1 and 5 min, perinatal depression (based on the needing for post-natal cooling therapy, or post-natal MRI showed hypoxic-ischemic encephalopathy), intrauterine growth restriction (based on regular antenatal ultrasound follow up), length of hospital stay, mortality, the need for resuscitation (defined as the need for cardiopulmonary resuscitation or epinephrine at any dose by endotracheal tube, umbilical venous catheters, or intravenous), and confirmation of early-onset sepsis (within 72 h of birth) and late-onset sepsis depending on positive cultures from cerebrospinal fluid and blood.

Other data on short-term morbidities, including neonatal jaundice (NNJ) which confirmed the need for NICU admission to manage NNJ in the first 72 hours of life; hypoglycemia depending on glucose level (less than 2.6 mmol/L); congenital anomalies from clinical findings, positive tests, and radiological findings; cardiac anomalies on echocardiography, performed when the infant developed hemodynamic instability and necessitated medical or surgical treatment; and renal anomalies from findings of renal ultrasound scans. In this study, central nervous system (CNS) anomalies were confirmed from a positive brain or spinal cord image using ultrasonography, computed tomography, and magnetic resonance imaging. A neonatal seizure was defined as an abnormal movement or need for anti-epileptic medication; respiratory distress syndrome (RDS) from the need for beractant; and congenital pneumonia from radiological findings, with a need for respiratory support and discharge diagnosis.

Data entry and analysis

Data entry was performed using SPSS v. 22.0 (IBM Corp., Armonk, NY) for all statistical analyses. Categorical data are presented as numbers and percentages, whereas continuous variables are presented as means and standard deviations. The association between morbidities and mortality risk was analyzed using multivariate regression analysis and odds ratios with 95% confidence interval (CIs). P-values <0.05 were considered significant. Frequencies were analyzed using the chi-squared test. Percentiles, medians, and associations between variables were analyzed using the regression model and Mann-Whitney test.

Ethical considerations

The Research Ethics Committee at the Unit of Biomedical Ethics, King Abdulaziz University, Faculty of Medicine, Jeddah, Saudi Arabia approved this retrospective study conducted at the KAUH in Jeddah. The study was approved by the institutional review board of the KAUH (reference number: 336-22). The study was conducted in accordance with the guidelines of the Declaration of Helsinki.

## Results

This study included 186 participants, with approximately two-thirds (69.9%) of them being of Saudi nationality. The maternal median age was 32 (interquartile range (IQR) 27-36) years, and the median number of pregnancies was three (IQR 1-5). Cesarean section (CS) was the most common mode of delivery (67.2%), followed by spontaneous vaginal delivery (32.8%). Most cesarean deliveries (78.7%) were emergency CS. The median gestational age at birth was 36 (IQR 34-38) weeks, with more than half of the mothers (69.4%) being booked. Comorbid chronic conditions were reported in 40.3% of mothers, including hypertension (14%), diabetes mellitus (20.4%), and hypothyroidism (10.8%). Only 6.5% of mothers reported previous baby admissions to the NICU, whereas nearly one-third (36%) received antenatal steroids (Table 1).

**Table 1:** Demographic and prenatal characteristics of mothers IQR, interquartile range; SVD, spontaneous vaginal delivery; CS, cesarean section; HTN, hypertension; DM, diabetes mellitus; NICU, neonatal intensive care unit

		N	N%	Median (IQR)
Nationality	Non-Saudi	56	30.1%	
	Saudi	130	69.9%	
Age in years				32 (27–36)
Gravidity				3 (1–5)
Mode of delivery	SVD	61	32.8%	
	CS	125	67.2%	
Mode of CS	Elective	26	21.3%	
	Emergency	96	78.7%	
Gestational age in weeks				36 (34–38)
Booking status	Not booked	57	30.6%	
	Booked	129	69.4%	
Maternal comorbidities	No	111	59.7%	
	Yes	66	40.3%	
Comorbid conditions	HTN	26	14.0%	
	DM	38	20.4%	
	Hypothyroidism	20	10.8%	
Previous baby admitted to the NICU	No	128	68.8%	
	Yes	12	6.5%	
Antenatal steroid	No	110	59.1%	
	Yes	67	36.0%	

Regarding the outcome of deliveries, approximately half (50.5%) were females. The median birthweight was 2325 (IQR 1740-2900) g. The median Apgar scores at 1 and 5 mins were 7 (IQR 5-9) and 9 (IQR 8-10), respectively. Most deliveries (78.5%) were singletons, whereas twin deliveries were the most prevalent among the remaining multiple pregnancies. Jaundice was the most common postnatal complication (51.6%), followed by hypoglycemia (18.8%). Nearly one-quarter (23.7%) of babies had congenital cardiac (70.5%), renal (18.2%), or CNS anomalies (29.5%). The median length of hospital stay was 10 (IQR 5-18) days, and the mortality percentage was 5.9% (Table 2).

**Table 2:** Post-natal delivery outcomes RDS, respiratory distress syndrome; CNS, central nervous system; IQR, interquartile range

		N	N %	Median (IQR)
Sex	Female	94	50.5%	
	Male	92	49.5%	
Birth weight in grams				2325 (1740–2900)
Apgar score at 1 min				7 (5–9)
Apgar score at 5 min				9 (8–10)
Multiple gestations	No	146	78.5%	
	Yes	40	21.5%	
Outcome of multiple gestations	Twins	32	80.0%	
	Triplets	8	20.0%	
Post-natal complications	Need for resuscitation	10	5.4%	
	Perinatal depression	3	1.6%	
	Neonatal seizure	9	4.8%	
	RDS	10	5.4%	
	Congenital pneumonia	5	2.7%	
	Sepsis	10	5.4%	
	Jaundice	96	51.6%	
	Hypoglycemia	35	18.8%	
Congenital anomalies	No	142	76.3%	
	Yes	44	23.7%	
Type of congenital anomalies	Cardiac anomalies	31	70.5%	
	Renal anomalies	8	18.2%	
	CNS anomalies	13	29.5%	
Length of hospital stay in days				10 (5–18)
Mortality	No	175	94.1%	
	Yes	11	5.9%	

Maternal booking status was related to several maternal and fetal factors. Booking status was associated with Saudi nationality (p < 0.001), higher maternal age (p = 0.007), emergency mode of CS (p = 0.008), DM and hypothyroidism (p = 0.022), multiple gestations of triplets (p = 0.036), and fewer instances of postnatal complications including jaundice (p = 0.036), hypoglycemia (p = 0.011), and need for resuscitation (p = 0.038) (Tables 3-4).

**Table 3:** Demographic and prenatal characteristics in correlation with booking status IQR, interquartile range; SVD, spontaneous vaginal delivery; CS, cesarean section; HTN, hypertension; DM, diabetes mellitus; NICU, neonatal intensive care unit *P-values <0.05 were considered significant

Demographic characteristics		Booking status				
		Not booked (N=57)		Booked (N=129)		p-value
		N (%)	Median (IQR)	N (%)	Median (IQR)	
Nationality	Non-Saudi	38 (66.7%)		18 (14%)		<0.001*
	Saudi	19 (33.3%)		111 (86%)		
Age in years			29 (23–34)		32 (28–37)	0.007*
Gravidity			3 (1–5)		3 (2–5)	0.984
Mode of delivery	SVD	20 (35.1%)		41 (31.8%)		0.658
	CS	37 (64.9%)		88 (68.2%)		
Mode of CS	Elective	2 (5.7%)		24 (27.6%)		0.008*
	Emergency	33 (94.3%)		63 (72.4%)		
Gestational age in weeks			36 (34–38)		37 (34–48)	0.135
Maternal comorbidities	No	36 (73.5%)		75 (58.6%)		0.067
	Yes	13 (26.5%)		53 (41.4%)		
Comorbid conditions	HTN	8 (61.5%)		18 (34%)		0.022*
	DM	5 (38.5%)		33 (62.3%)		
	Hypothyroidism	1 (7.7%)		19 (35.8%)		
Previous baby admitted to the NICU	No	39 (95.1%)		89 (89.9%)		0.315
	Yes	2 (4.9%)		10 (10.1%)		
Antenatal steroid	No	37 (69.8%)		73 (58.9%)		0.169
	Yes	16 (30.2%)		51 (41.1%)		

**Table 4:** Post-natal delivery outcomes in correlation with booking status RDS, respiratory distress syndrome; CNS, central nervous system; IQR, interquartile range * P-values <0.05 were considered significant.

Post-natal delivery outcomes		Booking status				
		Not booked (N=57)		Booked (N=129)		p-value
		N (%)	Median (IQR)	N (%)	Median (IQR)	
Sex	Female	24 (42.1%)		70 (54.3%)		0.126
	Male	33 (57.9%)		59 (45.7%)		
Birth weight in g			2220 (1740–2850)		2365 (1795–2900)	0.791
Apgar score at 1 min			7 (5–8)		8 (6–9)	0.434
Apgar score at 5 min			9 (8–10)		9 (8–10)	0.227
Multiple gestations	No	47 (82.5%)		99 (76.7%)		0.382
	Yes	10 (17.5%)		30 (23.3%)		
Outcome of multiple gestations	Twins	10 (100%)		22 (73.3%)		0.036*
	Triplets	0 (0%)		8 (26.7%)		
Post-natal complications	Need for resuscitation	6 (12.8%)		4 (4.7%)		0.038*
	Perinatal depression	2 (4.3%)		1 (1.2%)		0.172
	Neonatal seizure	1 (2.1%)		8 (9.3%)		0.193
	RDS	3 (6.4%)		7 (8.1%)		0.964
	Congenital pneumonia	1 (2.1%)		4 (4.7%)		0.601
	Sepsis	3 (6.4%)		7 (8.1%)		0.964
	Jaundice	36 (76.6%)		60 (69.8%)		0.036*
	Hypoglycemia	17 (36.2%)		18 (20.9%)		0.011*
Congenital anomalies	No	44 (77.2%)		98 (76%)		0.856
	Yes	13 (22.8%)		31 (24%)		
Type of congenital anomalies	Cardiac anomalies	11 (84.6%)		20 (64.5%)		0.319
	Renal anomalies	1 (7.7%)		7 (22.6%)		
	CNS anomalies	3 (23.1%)		10 (32.3%)		
Length of hospital stay in days			12 (7–19)		9 (5–17)	0.097
Mortality	No	54 (94.7%)		121 (93.8%)		0.802
	Yes	3 (5.3%)		8 (6.2%)		

Multivariate logistic regression analysis was used to examine the effect of maternal and fetal factors on fetal mortality outcomes. The overall model containing 13 potential predictors was significant (X2 (13, N=186) = 25.52, p = 0.02). Only two independent variables were significant predictors of fetal mortality (Table 5). Maternal age and fetal Apgar score at 5 min were significant negative predictors, with odds ratios of 0.755 and 0.096, respectively, indicating increased mortality with decreasing maternal age and Apgar score at 5 min.

**Table 5:** Maternal and fetal predictors of fetal mortality outcome Variable(s) entered: Maternal age, gravidity, gestational age, booking status, mode of delivery, chronic diseases, previous baby admitted to the NICU, antenatal steroid use, birthweight, Apgar score at 1 min, Apgar score at 5 min, sex, and length of hospital stay. CS: cesarean section; NICU: neonatal intensive care unit; CI: confidence interval; B: unstandardized regression weight, the coefficient for the constant; SE: standard error around the coefficient for the constant; df: degree of freedom; sig: significance; Exp (B): exponentiation of the B coefficient

Variables	B	S.E.	Wald	df	Sig.	Exp(B)	95% CI for Exp (B)	
							Lower	Upper
Maternal age	-0.281	0.13	4.69	1	0.03	0.755	0.585	0.974
Gravidity	0.198	0.44	0.203	1	0.652	1.219	0.515	2.89
Gestational age	-0.242	0.346	0.486	1	0.486	0.785	0.398	1.549
Maternal booking	1.891	1.328	2.026	1	0.155	6.625	0.49	89.523
CS mode of delivery	-2.149	1.298	2.74	1	0.098	0.117	0.009	1.485
Chronic diseases	-0.93	1.498	0.386	1	0.535	0.394	0.021	7.433
Previous baby admitted to the NICU	1.403	0.88	2.54	1	0.111	4.067	0.724	22.831
Antenatal steroid use	-0.393	1.354	0.084	1	0.772	0.675	0.048	9.586
Birth weight	-0.001	0.001	0.464	1	0.496	0.999	0.997	1.002
Apgar score at 1 min	1.007	0.595	2.87	1	0.09	2.738	0.854	8.783
Apgar score at 5 min	-2.344	1.079	4.718	1	0.03	0.096	0.012	0.795
Male sex	0.368	0.999	0.136	1	0.713	1.445	0.204	10.229
Length of hospital stay	0.008	0.012	0.485	1	0.486	1.008	0.985	1.032

## Discussion

The analysis of maternal characteristics showed a significant difference in age distribution between unbooked (median age of 29 years) and booked (median age of 32 years) mothers, which is in line with that in previous reports indicating that younger women were less adherent to ANC follow-up [7,13-15]. In addition, the unbooking status of pregnant women has been linked to low socioeconomic status [16-18].

This study revealed a significant difference in booking status regarding the mother’s nationality, where 86% of booked women were of Saudi nationality and 66.7% of unbooked women were of non-Saudi nationality. This finding may reflect key inequalities in access to healthcare that may be attributed to various reasons, including social, cultural, and financial factors.

No significant difference was noted in the rates of CS between mothers who were unbooked (64.9%) and those who were booked (68.2%), which is comparable to the findings of Tucker and colleagues [13]. However, the rate of emergency CS was much higher in unbooked mothers (94.3%) than in booked ones (72.4%). This discrepancy in the rates of emergency CS may be attributed to a lack of vigilance to existing medical problems that may have been aggravated, necessitating emergency intervention. The prevalence of maternal comorbid conditions, including DM and hypothyroidism, was higher among booked women than among unbooked women, except for hypertension. This may be due to the sense of apprehension experienced by women with known comorbidities during pregnancy.

Although the number of women accessing ANC has increased significantly in underdeveloped nations, only a small percentage of expectant mothers optimally utilize ANC with at least four ANC visits, with 72% starting their first visit after 12 weeks of pregnancy [19]. For instance, a report from Kenya found that only 58% of women had at least four ANC visits [14], whereas a study conducted in Somalia found that the rate was even lower, with 33% of pregnant women starting their first visit in the fourth month of pregnancy [15]. Thus, the World Health Organization emphasizes the necessity of concentrating on pregnant women who start their ANC late and have fewer visits than usual [20].

The fetal outcomes of newborns admitted to the NICU with respect to the development of postnatal complications significantly differed between unbooked and booked mothers regarding the development of jaundice, hypoglycemia, and need for resuscitation, with 76.6%, 36.2%, and 12.8% of unbooked mothers reporting these complications, compared with 69.8%, 20.9%, and 4.7% of booked mothers, respectively.

Fetal mortality was observed in only 5.9% of study participants, with no significant difference between mothers regarding booking status. This is similar to previously reported evidence, in which no significant difference in perinatal mortality was documented in mothers’ adherence to ANC follow-up [7].

Psychological and psychosocial issues in pregnant women have gained much attention during prenatal care because of epidemiological evidence for their incrimination in the development of postpartum depression [21], with evidence of psychosocial and psychological interventions playing a vital role in reducing the occurrence of postpartum depression [22]. However, psychological support has been shown to receive minute emphasis during ANC [5]. Our study reported perinatal depression in only three women (1.6 %) in the study cohort. This may stem from the psychosocial support received during ANC or through other social support systems in the community. The importance of social support for pregnant women has been alluded to by a study conducted in Saudi Arabia in addition to studies from Argentina, Cuba, and Thailand [23].

The study signifies that non-attendance of ANC is significantly associated with poorer pregnancy outcomes in the form of the need for neonatal resuscitation and the occurrence of neonatal jaundice and hypoglycemia. These results agree with the literature as these poor pregnancy outcomes are more common in unbooked pregnant women. In contrast, no significant association was found between the booking status of pregnant women and birthweight, Apgar score result, RDS, neonatal seizures, or neonatal mortality. These outcomes are different from previously reported results regarding the same factors, since they have stated that neonatal mortality, low birthweight, and other devastating pregnancy outcomes are more prevalent among unbooked pregnant women [7,24].

Regarding the fetal and maternal factors that influence fetal mortality, decreasing maternal age and lower fetal Apgar score at 5 min were the only factors that increased fetal mortality. Other studied factors were not significant predictors of fetal mortality. However, previous studies have indicated increasing maternal age as a significant factor for fetal mortality among others [7,24].

Limitations

This study was not without limitations. One limitation was the difficulty in accessing data, due to improper documentation in some cases, as well as cultural constraints in interviewing females in the area. Furthermore, a retrospective cross-sectional study design limits the credibility of inferences drawn from the data analysis. A cohort study design would have provided better information about maternal and fetal outcomes in relation to maternal ANC adherence.

## Conclusions

This study revealed suboptimal adherence to ANC practices among pregnant women whose children were admitted to the NICU. In addition, this study indicated poorer maternal and fetal outcomes in unbooked women, including emergency CS, neonatal jaundice, hypoglycemia, and the need for resuscitation. Moreover, predictors of fetal mortality included decreasing maternal age and Apgar score at 5 min.

We recommend that future researchers determine the underlying reasons for suboptimal adherence to ANC, which results in poorer outcomes regarding both fetuses and pregnant women. Additionally, it is recommended that policymakers establish educational campaigns to emphasize the importance of ANC to improve the overall outcomes of pregnancy.
